# Exploration and optimization of a respiratory outpatient triage model during the X disease epidemic: A retrospective observational study

**DOI:** 10.1097/MD.0000000000049150

**Published:** 2026-06-05

**Authors:** Chao Zhou, Xiao Feng, Hanhan Hong

**Affiliations:** a Department of Respiratory and Critical Care Medicine, Changzheng Hospital, Navy Medical University, Shanghai, China.

**Keywords:** emergency department, epidemic, infection control, outpatient clinic, patient severity, preexamination triage, respiratory infections, triage, triage accuracy, workflow optimization, X disease

## Abstract

Preexamination triage is the critical first line of defense in outpatient settings. During epidemics like the X disease outbreak, traditional models often fail, and inaccurate triage leads to unfavorable patient outcomes and infection risks. This study aims to address the lack of specific, optimized triage workflows for respiratory epidemics. A retrospective observational study was conducted during the “Harmonious Mission-2022” deployment. An optimized 3-tier triage system – Entrance Security Check, Antigen Testing Area, and Preexamination Triage Area – integrated with a 3-level severity classification was implemented. Primary outcomes were triage efficiency, defined as mean daily patient throughput, and triage accuracy, defined as concordance between initial triage assessment and final diagnosis or disposition. Data were analyzed using descriptive statistics, and operational constraints were identified through structured observational checklists. The optimized model processed a mean daily volume of 550.43 (standard deviation ± 284.13) individuals and achieved a triage accuracy rate of 98.3%. Key limiting factors included language barriers and environmental noise, which were addressed through standardized protocols and functional zoning. The structured 3-tier model demonstrated high operational efficiency and diagnostic concordance during the epidemic surge. These findings support the potential value of tiered task separation in strengthening preparedness for future respiratory outbreaks.

## 1. Introduction

During major public health emergencies, particularly the epidemic of X disease, outpatient and emergency (O&E) medical areas face immense pressure. X disease is a highly contagious respiratory illness characterized by rapid transmission and a wide spectrum of clinical severity. Its outbreak precipitated a dramatic surge in patient volume that far exceeded routine capacity, necessitating urgent alterations to standard workflows. The convergence of large patient numbers, case complexity, and the critical need for specific infectious disease screening makes effective preexamination triage paramount. Triage serves as the frontline process for sorting patients based on urgency, prioritizing care for the most critically ill. An accurate and efficient triage system is recognized as a cornerstone strategy for improving the allocation of limited healthcare resources, mitigating the severe overcrowding prevalent in emergency settings, and enhancing the overall quality of patient care.^[1,2]^

Prior to the epidemic, the standard workflow relied primarily on simple administrative sorting at the reception desk, lacking specific infectious disease screening layers or distinct physical zoning. However, this traditional model proved inadequate during the outbreak. Deficiencies in the triage process can lead to significant negative consequences. Specifically, the absence of separate pathways increased the risk of nosocomial cross-infection, while the simplified sorting process caused severe operational bottlenecks. Patient dissatisfaction stemming from perceived delays, lack of clarity, or unfair prioritization can erode trust in healthcare institutions and potentially lead to increased consumption of hospital resources.^[3]^ Furthermore, triage accuracy is influenced by a complex interplay of variables, including patient comorbidities, environmental stressors such as high ambient noise levels and overcrowding, and personnel factors including clinical experience and linguistic competency.^[4,5]^

Driven by the urgent necessity to address these operational failures and the limitations of the traditional workflow, this study was undertaken based on practical experience gained during the preexamination triage operations for the X disease epidemic. The primary objective was to meticulously analyze the implemented workflow, identify specific operational bottlenecks, and develop a structured, optimized triage model. The intention is for this refined model to serve as a practical reference and valuable guide for improving triage operations during future outbreaks of X disease or similar respiratory epidemics.

## 2. Methods

### 2.1. Study design and setting

This study utilized a retrospective observational design to evaluate the effectiveness of a triage optimization model. The study was conducted at the O&E medical center established during the “Harmonious Mission-2022” deployment. This setting was characterized by a temporary, high-volume medical facility operating in a resource-constrained environment during the epidemic of X disease, defined as a severe respiratory infectious disease. The site was selected as a suitable location for this study because it faced the dual challenges of a surge in patient volume and the requirement for strict infectious disease control, providing an ideal “stress test” environment for evaluating the optimized triage workflow.^[6]^

### 2.2. Study population

The study population included all patients who presented to the O&E medical area seeking medical attention during the mission period. Patients included local residents exhibiting various medical needs, ranging from minor ailments to severe respiratory symptoms consistent with X disease. Individuals entering the area solely for nonmedical purposes, such as logistics delivery personnel who did not undergo the full medical triage process, were excluded from the analysis.

### 2.3. Sample size and sampling considerations

The sample size consisted of the total patient volume processed during the “Harmonious Mission-2022” deployment. As this was a retrospective analysis of the entire operational period, no specific probability sampling method was employed; rather, a census of all patient visits during the defined study period was analyzed to ensure a comprehensive evaluation of the triage system’s performance.

### 2.4. Study procedures

#### 2.4.1. Triage workflow overview

Prior to the implementation of the optimized model, the workflow followed a traditional single-point entry system. To address the epidemic challenges, an optimized 3-tier triage system was developed and implemented. This system spatially and functionally divided the triage process into 3 distinct zones:

Tier 1: Entrance Security Check: Managed by a supervising nurse. Responsibilities included distributing informational health pamphlets, ensuring patients wore N95 masks correctly, and performing a “rapid visual screen” to identify obviously critical patients. Key indicators for immediate prioritization included signs of somnolence, severe pain, respiratory distress, or extreme frailty. High-risk patients were prioritized for immediate transfer to the resuscitation area.^[7]^

Tier 2: Antigen Testing Area: Staffed by 2 nurses. Their primary role was conducting rapid antigen testing for X disease and issuing clinic registration cards. Patients testing positive were immediately diverted to a designated isolation tent for management by a physician using Level 2 protective measures.

Tier 3: Preexamination Triage Area: This core assessment area was staffed by a team leader, 2 triage officers, and 1 medical assessor (senior clinician).

Triage officers conducted focused history taking and selected the appropriate clinical department.

The medical assessor actively monitored the queue for deteriorating patients, initiated stabilization for those unsuitable for outpatient management, and managed emergency events.

The team leader coordinated overall flow and liaised with the command center.^[8]^

#### 2.4.2. Patient severity classification

Patients were categorized into 3 levels based on urgency, adapted from established principles^[7]^: Level I (Critical/Resuscitation), Level II (Urgent/Priority), and Level III (Nonurgent). See Table 1 for detailed classification criteria.

**Table 1:** Patient severity classification.

Level	Description	Examples
I	Patients with severe conditions requiring immediate intervention.	Cardiac arrest, acute myocardial infarction, acute heart failure, acute respiratory failure, acute stroke, and sepsis.
II	Patients with serious conditions but currently stable vital signs.	Fractures in noncritical locations, minor head injury, and mild asthma exacerbation.
III	Patients with common, nonurgent conditions.	Upper respiratory tract infections, gastrointestinal infections, joint pain, skin conditions, vision decline, gynecological/pediatric issues, and suture removal/dressing change.

### 2.5. Data collection

#### 2.5.1. Quantitative data

Data regarding triage efficiency and accuracy were extracted from the electronic medical records system and daily operational logs maintained by the central command group.

Efficiency data: Daily patient throughput numbers were recorded automatically by the registration system at Tier 2 and Tier 3.

**Figure 1.**
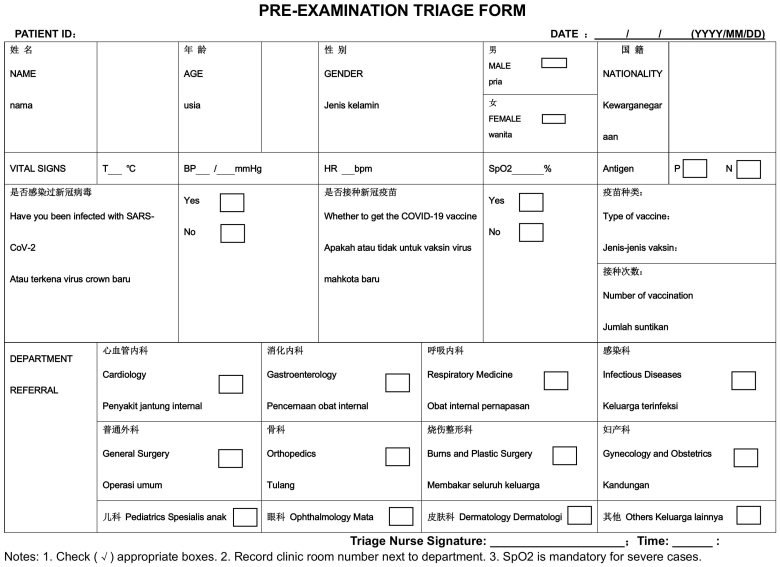
The standardized preexamination triage form utilized during the deployment. The form captures patient demographics, vital signs including mandatory SpO2 for severe cases, COVID-19 screening (antigen test and vaccination history), and specific clinical departments for referral.

Accuracy data: The initial triage categorization (Level and Department) recorded on the triage form (Fig. 1) was cross-referenced with the final diagnosis and disposition recorded by the treating physician in the electronic medical records.

#### 2.5.2. Qualitative data

To identify factors affecting triage efficacy, a structured observational checklist was utilized. This checklist was completed daily by the triage team leader and the nursing supervisor. It captured data on 3 primary domains: patient factors including communication difficulties; environmental factors such as noise levels and crowding; and personnel factors including fatigue and equipment issues. The specific items of the checklist are detailed in File S1, Supplemental Digital Content.

### 2.6. Data analysis

#### 2.6.1. Computation of efficacy and accuracy

Triage efficiency: Calculated as the mean daily throughput, defined as the total number of patients processed divided by the number of operational days.

Triage accuracy: Calculated using the formula: (Number of patients correctly triaged/Total number of patients triaged) × 100%. “Correctly triaged” was defined as concordance between the initial triage severity assessment/specialty assignment and the final clinical diagnosis/disposition.

#### 2.6.2. Qualitative analysis

Findings from the observational checklists were analyzed using thematic analysis. Daily entries were reviewed and categorized into 3 primary domains: patient factors, environmental factors, and personnel factors. These qualitative findings were used to contextualize the quantitative results and identify specific bottlenecks.

#### 2.6.3. Statistical methods

All data were analyzed using SPSS Statistics version 26.0 (IBM Corp). Descriptive statistics, specifically mean ± standard deviation for continuous variables and frequencies with percentages for categorical variables, were used to summarize the study findings. The results are presented in the following order: triage performance outcomes (efficiency and accuracy), followed by the analysis of factors influencing triage.

### 2.7. Ethical considerations

This study was conducted in accordance with the Declaration of Helsinki. The study protocol was approved by the Institutional Review Board of Changzheng Hospital. Due to the retrospective nature of the study and the use of de-identified data, the requirement for individual informed consent was waived by the Institutional Review Board.

## 3. Results

### 3.1. Triage performance outcomes

The implementation of this optimized 3-tier triage model during the “Harmonious Mission-2022” deployment yielded positive results. The system effectively facilitated the rapid identification of critically ill patients, enabling prompt initiation of rescue measures while successfully maintaining infection control standards. A marked improvement in triage workflow efficiency was observed, with the system processing an average of 550.43 (±284.13) individuals per day, corresponding to over 1100 patient visits daily. Based on assessment during this mission, the overall triage accuracy rate achieved was 98.3%.

### 3.2. Factors affecting triage efficiency and implemented optimizations

The analysis, based on data from observational checklists and staff debriefings as described in Section 2, identified several key factors impacting triage efficiency. These findings are summarized in Table 2 and further detailed below.

**Table 2:** Factors affecting triage efficiency and implemented optimizations.

Factor category	Issue description	Optimization recommendation/Strategy
Patient factors	• *Elderly patients with complex comorbidities*: Difficulty in articulating the primary complaint clearly. • *Language barriers*: Patients with limited proficiency in local languages could not describe symptoms effectively.	• *Focused assessment*: Staff utilized active listening and nonverbal cues to identify the main issue. • *Language support*: Deployment of medically knowledgeable translators and provision of short-term training on common medical terminology.
Environmental factors	• *Overcrowding and noise*: High patient volume created a chaotic environment that hindered communication and assessment. • *Resource limitations*: Constraints on available medical resources impacted the triage process.	• *Flow control*: Established a streamlined patient flow and used volunteers to manage queues. • *Severity-based prioritization*: Reinforced strict prioritization based on clinical urgency to optimize resource use.
Personnel factors	• *Time constraints*: High throughput reduced assessment time per patient, increasing the potential for error. • *Increased workload*: The need to measure vital signs for severe patients extended triage time, slowing overall flow.	• *Expert staffing*: Assigned senior clinicians with extensive experience and strong decision-making skills to key roles. • *“Severe first, stable fast” strategy*: Adopted a dynamic approach to process critical patients immediately while expediting less acute cases.

*Patient individual factors*:

- *Issue:* Elderly patients often presented with multiple chronic conditions, making it difficult for them to articulate their primary complaint clearly. A lack of medical knowledge hindered systematic symptom description.
- *Optimization:* Triage staff were encouraged to familiarize themselves with common geriatric presentations, utilize observation and nonverbal cues, practice active and patient listening to identify the main issue,^[9]^ and prioritize accurately based on the most critical problem.
- *Issue:* Language barriers arose with patients having limited proficiency in Chinese or English. Some available translators lacked experience in medical terminology, failing to capture the full scope of the patient’s needs.
- *Optimization:* Short-term language training focusing on common medical consultation terms relevant to the patient population was recommended. Experienced, medically knowledgeable translators were assigned to assist when available.^[10]^

*Environmental factors*:

- *Issue:* The high overall workload, resulting in overcrowding and a noisy environment, combined with limitations in available medical resources, negatively impacted the triage process.^[4,5]^
- *Optimization:* Efforts were made to establish a more streamlined patient flow. Volunteers were utilized to help manage queues and maintain order. Triage prioritization based strictly on severity was reinforced.

*Triage personnel factors*:

- *Issue:* The sheer volume of patients reduced the time available for each assessment, potentially increasing the risk of triage errors. The need to measure vital signs (blood pressure and oxygen saturation) for more severe patients inevitably extended triage time for those individuals, slowing overall throughput and potentially impacting safety if delays occurred.^[7,11]^
- *Optimization:* Staffing for triage roles prioritized senior medical and nursing personnel possessing extensive clinical experience, strong communication skills, and rapid decision-making abilities. A triage strategy emphasizing “severe first, stable fast” was adopted, where critical patients received immediate attention while less acute cases were processed more rapidly.^[8,12]^

### 3.3. Analysis and management of healthcare staff and patient concerns

In addition to operational factors, the analysis identified key concerns from both healthcare providers and patients. These concerns, along with the corresponding solutions implemented to address them, are summarized in Table 3. Concerns from both healthcare providers and patients were identified:

**Table 3:** Analysis of staff and patient concerns and implemented interventions.

Stakeholder group	Identified concerns	Implemented solutions/Interventions
Healthcare staff	• *Fear of infection*: Anxiety regarding personal infection risk due to limitations in Personal Protective Equipment (PPE). • *High triage pressure*: Stress from high patient volume, media attention, and risk of workplace violence. • *Impact on well-being*: Reports of insomnia, fatigue, and increased irritability due to high workload.	• *Health & safety*: Provided proactive health support (vaccinations, monitoring) and intensified training on infection control protocols. • *Psychological & leadership support*: Offered regular support from leadership and framed the work as a professional growth opportunity to boost morale. • *Communication training*: Emphasized training on empathetic communication to de-escalate conflicts.
Patients	• *Triage environment*: Concerns about lack of privacy and confidentiality for sensitive issues due to limited space. • *Healthcare provider factors*: Fear of delayed treatment due to long waits, confusion about the triage process, and perceived negative staff attitudes.	• *Environmental adjustments*: Increased spacing between patients and placed clear informational signboards at each triage point. • *Process reassurance*: Staffed triage points with experienced clinicians to ensure accurate prioritization and deployed volunteers to answer questions and provide guidance. • *Service orientation*: Ensured staff maintained a warm, respectful, and service-oriented attitude.

- *Healthcare staff concerns*:

1. *Fear of infection and transmission:* Due to local policies preventing the use of full Level 2 personal protective equipment, staff (wearing N95 masks, caps, and basic eye protection) expressed anxiety about personal infection risk and potential cross-contamination within the facility.
2. *High triage pressure:* Staff reported pressure from multiple sources: public and media attention creating performance anxiety; operational stress due to high patient volume, relative staff shortages, and communication difficulties; and the perceived risk of workplace violence (verbal abuse and physical aggression being common, often linked to long wait times or communication breakdowns).^[11]^
3. *Impact on well-being:* The combination of high workload intensity and various pressures led to reports of adverse effects on mental and physical health, including insomnia, fatigue, and increased irritability.^[13]^

- *Patient concerns*:

1. *Triage environment:* Patients expressed concerns about the limited space in the triage area, leading to a lack of privacy and confidentiality, particularly for sensitive issues (e.g., gynecological complaints).
2. *Healthcare provider factors:* Key concerns included fear that prolonged waiting times could delay necessary diagnosis and treatment; confusion about the process and next steps after entering the triage area; and dissatisfaction with perceived negative attitudes from staff.^[3]^

### 3.4. Implemented solutions for addressing concerns

Strategies were implemented to mitigate these concerns:

- *For healthcare staff*: First, proactive health support including access to free vaccinations (e.g., relevant vaccines), immunomodulators (e.g., Thymosin alpha 1), regular screening for respiratory symptoms (fever and cough), and daily temperature monitoring. Second, intensified training on infectious disease protection protocols and repeated simulation drills of the complete emergency plan to build confidence.^[13]^ Third, encouragement for staff to view the challenging work as an opportunity for professional growth, skill development, and gaining valuable experience. Fourth, regular expressions of support, care, and formal recognition from organizational leadership (including Party organizations) to boost morale. Finally, Emphasis on training for effective and empathetic communication with patients to understand their perspectives, build rapport, and potentially de-escalate tense situations, thereby reducing the risk of conflict.^[11]^
- *For patients*: First, physical adjustments to increase spacing between waiting patients where possible. Triage staff were reminded to be mindful of patient privacy during questioning. Second, reassurance provided by staffing triage points with experienced clinicians capable of accurate, orderly prioritization and rapid identification of high-risk patients needing expedited care.^[8]^ Third, placement of clear informational signboards at each triage point, supplemented by volunteers available to explain the process and answer questions. Finally, ensuring staff maintained a service-oriented, warm, and respectful attitude, adhering to a people-centered approach.

## 4. Discussion

The primary aim of this study was to analyze an existing preexamination triage workflow and evaluate an optimized model designed for the specific challenges of the X disease epidemic. Our findings demonstrate 3 key outcomes: the optimized 3-tier model achieved high triage efficiency, successfully managing a large surge in patient volume; it maintained a high degree of triage accuracy (98.3%); and the study systematically identified critical factors influencing triage outcomes, including patient, environmental, and personnel variables, which were addressed through targeted interventions.^[8,14]^

Our model demonstrated substantial operational efficiency, processing a mean of 550.43 individuals daily. This high throughput is critical during an epidemic surge, where the primary goal is to prevent the healthcare system from becoming overwhelmed. While direct comparison with routine emergency department throughput is difficult due to differing contexts, the model’s capacity to prevent systemic collapse and maintain orderly patient flow is a key indicator of its effectiveness. The structural separation of tasks – specifically, isolating the antigen testing process (Tier 2) from clinical assessment (Tier 3) – was instrumental in preventing bottlenecks and ensuring a continuous, parallel workflow. This finding underscores the novelty of designing triage systems not just for clinical accuracy but also for logistical resilience in high-volume, infection-risk settings.

The triage accuracy rate of 98.3% achieved in this study is notably high. For comparison, studies evaluating established systems like the Canadian Triage and Acuity Scale^[15]^ often report accuracy rates ranging from 85% to 95% in routine settings. We attribute our high accuracy primarily to the strategic placement of senior clinicians in the “Medical Assessor” role at the triage front line, a departure from many standard protocols that rely on junior nursing staff for initial assessment. This result suggests that embedding experienced clinical decision-makers directly into the triage process is a powerful intervention to minimize both under-triage and over-triage, thereby improving patient safety and resource allocation. This study provides strong evidence that investing senior personnel at the point of first contact is a high-yield strategy in crisis response.

Furthermore, this study systematically identified and addressed factors influencing triage outcomes. While challenges such as language barriers and patient comorbidities are well-documented in standard triage literature,^[16]^ their impact is significantly amplified in an epidemic context characterized by fear, high patient volume, and communication barriers imposed by personal protective equipment.^[17]^ Our study’s contribution lies in linking these identified factors directly to tested optimization strategies within a real-world crisis scenario, such as deploying medically knowledgeable translators and implementing a “severe first, stable fast” workflow. This provides a practical, evidence-based framework that other institutions can adapt, moving beyond theoretical knowledge to applied operational science.

This study has several strengths. First, the deployment of senior medical experts in key triage roles, rather than relying solely on administrative or junior staff, significantly enhances the validity and credibility of the triage decisions and the high accuracy rate reported. Second, the model was developed and tested in a real-world, high-pressure epidemic setting (“Harmonious Mission-2022”), ensuring its practical applicability and relevance. The systematic use of observational checklists provided rich qualitative data that contextualized and explained the quantitative performance outcomes.

Despite these strengths, several limitations must be acknowledged. First, this was a single-center study conducted within a specific mission-based medical deployment; therefore, the findings may not be directly generalizable to all traditional hospital emergency departments with different resources and patient populations. Second, the retrospective design relies on the accuracy of existing documentation and prevents the establishment of causality. Third, the qualitative data captured by the observation checklists were completed by internal team leaders and supervisors, which introduces a potential for reporting bias. Although daily debriefings were used to corroborate findings, the subjectivity of this data source remains a limitation.

## 5. Conclusion

This study detailed the exploration and optimization of a preexamination triage model specifically designed for respiratory outpatient clinics during the X disease epidemic. A structured 3-tier system combined with a 3-level patient severity classification was developed and evaluated. The optimized model demonstrated high operational efficiency and high triage accuracy, achieving a mean daily throughput of 550.43 ± 284.13 individuals and an accuracy rate of 98.3%, while effectively addressing patient, environmental, and personnel factors influencing triage performance. Overall, these findings indicate that a tiered structure with clear task separation and senior clinician involvement enhances surge management and patient safety during epidemic conditions. Future multi-center studies are needed to further validate the model and address limitations related to retrospective design and potential reporting bias.

## 6. Recommendations

Based on our findings, we propose the following recommendations. For clinical practice, healthcare facilities preparing for future epidemics should consider adopting a tiered zoning approach to separate infectious screening from clinical assessment and embed senior clinicians in frontline triage roles. For future research, multi-center studies are needed to validate this model in different settings. Furthermore, future work should explore the integration of information technology and artificial intelligence tools to automate aspects of the triage process, potentially reducing human error and alleviating pressure on staff during surges.

## Author contributions

**Conceptualization:** Xiao Feng, Hanhan Hong.

**Data curation:** Xiao Feng.

**Formal analysis:** Xiao Feng.

**Writing – original draft:** Chao Zhou, Xiao Feng.

**Writing – review & editing:** Chao Zhou, Hanhan Hong.



## References

[1] Moon SH Shim JL Park KS Park C-S. Triage accuracy and causes of mistriage using the Korean Triage and Acuity Scale. PLoS One. 2019;14:e0216972. 31490937 10.1371/journal.pone.0216972 PMC6730846

[2] Zhao XL Qiao AH Chai S. Application experience of secondary triage working mode in overseas medical outpatient service of “Peace Ark-2018” hospital ship [in Chinese]. J Navy Med. 2019;40:205–7.

[3] Phiri M Heyns T Coetzee I. Patients’ experiences of triage in an emergency department: a phenomenographic study. Appl Nurs Res. 2020;54:151271. 32650888 10.1016/j.apnr.2020.151271

[4] Ausserhofer D Zaboli A Pfeifer N. Errors in nurse-led triage: an observational study. Int J Nurs Stud. 2021;113:103788. 33120136 10.1016/j.ijnurstu.2020.103788

[5] Nguyen H Meczner A Burslam-Dawe K Hayhoe B. Triage errors in primary and pre-primary care. J Med Internet Res. 2022;24:e37209. 35749166 10.2196/37209 PMC9270711

[6] Zou Y Teng F Zhang RQ. Pre-examination triage model of maritime hospital [in Chinese]. J PLA Hosp Manage. 2020;27:130–5.

[7] Yuzeng S Hui LL. Improving the wait time to triage at the emergency department. BMJ Open Qual. 2020;9:e000708. 10.1136/bmjoq-2019-000708 PMC7011881 32019749

[8] Christian MD. Triage. Crit Care Clin. 2019;35:575–89. 31445606 10.1016/j.ccc.2019.06.009 PMC7127292

[9] Wang ZX Li H. Analysis of causes and countermeasures of triage errors in medical services of hospital ship in 5 Asian and African countries [in Chinese]. J Navy Med. 2011;32:82–3.

[10] Zhang CJ Li L Zhu HM. Analysis of factors affecting the efficiency and accuracy of wharf triage during overseas medical services performed by hospital ship and improvement measures [in Chinese]. J Navy Med. 2020;41:640–1.

[11] Reblora JM Lopez V Goh YS. Experiences of nurses working in a triage area: an integrative review. Aust Crit Care. 2020;33:567–75. 32143883 10.1016/j.aucc.2020.01.005

[12] Reay G Smith-MacDonald L Then KL Hall M Rankin JA. Triage emergency nurse decision-making: incidental findings from a focus group study. Int Emerg Nurs. 2020;48:100791. 31494074 10.1016/j.ienj.2019.100791

[13] Xu S Yang Q Xie M Wang J Shan A Shi F. Work experience of triage nurses in emergency departments during the prevalence of COVID-19. Int Emerg Nurs. 2021;56:101003. 33866257 10.1016/j.ienj.2021.101003 PMC7953436

[14] Zhang LY Lang HJ Xu L. Research progress of emergency pre-examination triage system and information management [in Chinese]. Chin Nurs Res. 2020;34:4414–9.

[15] Bullard MJ Musgrave E Warren D. Revisions to the Canadian Emergency Department Triage and Acuity Scale (CTAS) guidelines 2016. CJEM. 2017;19:S18–27. 28756800 10.1017/cem.2017.365

[16] Müller F Schröder D Noack EM. Overcoming language barriers in paramedic care with an app designed to improve communication with foreign-language patients: nonrandomized controlled pilot study. JMIR Form Res. 2023;7:e43255. 36951895 10.2196/43255 PMC10131716

[17] Díaz-Agea JL Orcajada-Muñoz I Leal-Costa C. How did the pandemic affect communication in clinical settings? A qualitative study with critical and emergency care nurses. Healthcare (Basel). 2022;10:373. 35206987 10.3390/healthcare10020373 PMC8872094

