# Supplementary material for: Exploration and optimization of a respiratory outpatient triage model during the X disease epidemic: A retrospective observational study
Source: Medicine (Baltimore). 2026 Jun 5;105(23):e49150. doi: 10.1097/MD.0000000000049150 (PMC13246078; doi:10.1097/MD.0000000000049150)
Supplement: Supplementary file 1 [file medi-105-e49150-s001.docx]

**Date:** _________________________

**Observer's Name/ID:** _________________________

**Shift Period (e.g., Morning, Afternoon, Night):** _________________________

**Part 1: Environmental Factors**

| **Item** | **Check (✔)** | **Notes & Specific Observations (Provide details if an issue is identified)** |
| --- | --- | --- |
| **1.1 Overcrowding:** Is the triage waiting area excessively crowded, impeding patient flow? | □ Yes □ No |  |
| **1.2 Noise Level:** Is the ambient noise level high enough to interfere with staff-patient communication? | □ Yes □ No |  |
| **1.3 Privacy:** Is there adequate physical space to ensure patient privacy during history-taking? | □ Yes □ No |  |
| **1.4 Resource Availability:** Are essential supplies (e.g., wheelchairs, thermometers, forms) readily available? | □ Yes □ No |  |

**Part 2: Patient Factors**

| **Item** | **Check (✔)** | **Notes & Specific Observations (Estimate number of patients affected if possible)** |
| --- | --- | --- |
| **2.1 Language Barriers:** Were there instances of significant communication difficulty due to language differences? | □ Yes □ No |  |
| **2.2 Complex Comorbidities:** Did elderly or chronically ill patients have significant difficulty articulating their primary complaint? | □ Yes □ No |  |
| **2.3 Patient Anxiety/Agitation:** Were there notable instances of patient distress or conflict related to wait times or communication? | □ Yes □ No |  |

**Part 3: Triage Personnel Factors**

| **Item** | **Check (✔)** | **Notes & Specific Observations (Describe impact on workflow)** |
| --- | --- | --- |
| **3.1 Workload:** Does the patient volume appear to exceed the staff's capacity for timely assessment? | □ Yes □ No |  |
| **3.2 Staff Fatigue:** Are there visible signs of physical or mental fatigue among the triage team members? | □ Yes □ No |  |
| **3.3 Procedural Adherence:** Is the team consistently following all steps of the triage protocol (e.g., PPE, documentation)? | □ Yes □ No |  |
| **3.4 Inter-team Communication:** Is communication between triage tiers and with downstream departments clear and effective? | □ Yes □ No |  |

**Part 4: Overall Summary & Actionable Recommendations**

**Briefly summarize the main challenges observed during this shift:**
________________________________________________________________________________
________________________________________________________________________________

**Immediate actions taken or recommended (e.g., requested additional volunteers, re-arranged waiting area):**
________________________________________________________________________________
________________________________________________________________________________
